# Comparison of Metabolomic Signatures Between Low and Heavy Parasite Burden of *Haemonchus contortus* in Meat Goats Fed with *Cynodon dactylon* (Bermudagrass) and *Crotalaria juncea* L. (Sunn Hemp)

**DOI:** 10.3390/metabo15110741

**Published:** 2025-11-14

**Authors:** Mariline Hilaire, Brandon Gines, Willard E. Collier, Honghe Wang, Santosh Chaudhary, Vivian Kanyi, Heba Abdo, Hossam Ismael, Erick Cathsley St. Preux, Melissa Boersma, Byeng R. Min

**Affiliations:** 1 Department of Agricultural and Environmental Sciences, Tuskegee University, Tuskegee, AL 36088, USA; mhilaire6334@tuskegee.edu (M.H.); schaudhary8992@tuskegee.edu (S.C.); vkanyri1503@tuskegee.edu (V.K.); habdo@tuskegee.edu (H.A.); hismael@tuskegee.edu (H.I.); estpreux5585@tuskegee.edu (E.C.S.P.); 2 Department of Chemistry, Tuskegee University, Tuskegee, AL 36088, USA; wcollier@tuskegee.edu; 3 Department of Biology, Tuskegee University, Tuskegee, AL 36088, USA; hwang@tuskegee.edu; 4 Mass Spectrometry Lab, Department of Chemistry and Biochemistry, Auburn University, Auburn, AL 36849, USA; mdb0067@auburn.edu

**Keywords:** goats, *Haemonchus contortus*, parasite, FEC, ^1^H-NMR and LC/MS, metabolomics, metabolic pathways

## Abstract

**Background/Objectives:** Animal health remains a critical issue that directly impacts economic sustainability through animal welfare and production. In small ruminants, the gastrointestinal parasite *Haemonchus contortus* can lead to anemia and possibly mortality, since parasite burden can be considerable and is challenging to control. Small ruminant health can be affected by poor diet and environmental conditions that lead to changes in the metabolic balance. The link between animal health and metabolic profiles has been investigated in the past. These studies have shed important light on physiological changes by identifying dietary and disease biomarkers. This study aimed to correlate the metabolite signature of feces from goats, having two levels of *Haemonchus contortus* infection, grazing on two different forages (Bermudagrass and Sunn Hemp). **Methods:** Fecal samples were taken from goats grazing either Sunn Hemp or Bermudagrass pastures, with naturally variable *Haemonchus contortus* loads. Samples were evaluated using ^1^H-NMR and LC/MS methods to describe and compare metabolic patterns under varied forage conditions between low and high Fecal Egg Count (FEC). **Results:** Our findings indicated no significant difference using univariate analyses but identified 10 discriminatory features using multivariate analyses for Bermudagrass and Sunn Hemp using ^1^H-NMR. With LC-MS, we found 14 significantly different features (*p* < 0.05, FC > 2), 115 discriminatory features for Bermudagrass and 113 in Sunn Hemp from multivariate analyses. Combining the findings of the two approaches suggested that *Haemonchus contortus* influenced several pathways associated with the metabolism of amino acids and energy conversion. **Conclusions:** The analysis of metabolome changes across both forages may help in revealing novel knowledge and accurate identification of possible biomarkers for gastrointestinal parasites. Further study is needed to validate the potential biomarker before deploying diagnostic tools based on the metabolomics indicators for early parasite diagnosis.

## 1. Introduction

Metabolic profiling is increasingly popular as a technique to identify changes in metabolism induced by diet adaptations and to distinguish the effects of variations in the gut flora on metabolism. Matysik et al. profiled human and murine fecal samples, which are abundant in unabsorbed metabolites, to provide a clearer indication of the metabolic interactions between gut microbiota and host [1]. Although metabolomics in urine, plasma, and tissue biopsies have been extensively investigated by researchers, fecal sample analysis, particularly concerning ruminants, has received relatively less attention [1,2]. Untargeted metabolomics allows scientists to impartially identify and characterize an organism’s entire range of endogenous small molecules. This approach uses bioinformatics to track changes brought on by disruptions such as genetic changes or environmental factors, as well as to uncover general metabolic trends [3,4]. Similarly, Tran, McConville & Loukopoulos reported that the metabolomic approach provides useful tools for detecting diseases, and biomarkers in animals [5].

Compounds derived from natural sources, especially plants, are promising alternatives for current anthelmintic drugs [6,7,8]. Many strategies have been explored to mitigate the economic damages of parasite infestation on small ruminants, including rotational grazing with different types of forages (including legumes such as Sunn Hemp and mixed forages), additive compounds, and natural dewormers to reduce parasitic burden [9]. In line with these strategies, feeding animals with Bermudagrass (*Cynodon dactylon*) and Sunn Hemp (*Crotalaria juncea* L.) may provide novel chemical properties for managing gastrointestinal nematodes and reducing fecal egg count in small ruminants. The increased demand for safer yet effective natural alternatives to synthetic anthelmintic drugs is driving research into alternative forages and their bioactive compounds to reduce parasite burden [10]. Moreover, rumen microbiota is significant for hosting different types of microorganisms such as fungi, bacteria, methanogens, and other species, many necessary for the digestion of feed, energy production, and the general health of livestock [11]. The existence of microorganisms found in the rumen allows the host to break down feedstuffs and digest different types of feed through microbial fermentation [12].

Metabolomics has become a powerful approach for identifying and quantifying low molecular-weight metabolites in biological samples [13]. It now plays a crucial role in many research areas, including food science, biomedical applications, and animal health [2]. For example, specific metabolites have been identified that influence the gut microbiome differently across several variables such as diet, age, and gender [14]. Metabolomics is one of the pivotal ways to identify novel biomarkers and biological pathways for quick, non-invasive diagnosis of diseases and monitoring of livestock health [15,16]. Metabolomics’ analytical procedures for biomarker identification have allowed scientists to tackle complex problems, including vigorous phenotype characterization of humans, animal breeding, crop plants, and pesticide monitoring [17]. Metabolomics creates a platform for the early detection of diseases and the identification of metabolic pathways for downstream transcriptomics and genomics [18]. This study has applied liquid chromatography-mass spectroscopy (LC/MS) and proton nuclear magnetic resonance spectroscopy (^1^H-NMR) to identify potential biomarkers associated with Haemonchosis infection in goat feces. LC/MS and ^1^H-NMR are the two common techniques for clinical diagnostics using metabolomics and have been used to analyze various biomatrices in domestic animals, such as feces, serum, and urine [13]. To our knowledge, no research has examined the differences in metabolomic signatures between low and heavy burdens of *Haemonchus contortus* in goats. This lack of detailed metabolomic profiles for different parasitic burdens warrants further investigations that could lead to new diagnostic tools for better parasite management. The objective of this research was to correlate the metabolome signature of feces in goats grazing on two different forages (Bermudagrass and Sunn Hemp) with high and low levels of *Haemonchus contortus* infection.

## 2. Materials and Methods

This research was carried out during the summer of 2024 at the George Washington Carver Experimental Station Farm in Tuskegee, Alabama. The experiment protocol was approved by the Tuskegee University Animal Care and Use Protocol Form R08-2024-06 before experimentation. Goats were allocated into two different pastures, Bermudagrass (*Cynodon dactylon*) and Sunn Hemp (*Crotalaria juncea* L.). This study examined the effects of grazing on Sunn Hemp (*Crotalaria juncea* L.) and Bermudagrass (*Cynodon dactylon*) on meat goat performance, fecal egg load, and metabolic profiles using four replicated plots. Forty-five (*n* = 45) Spanish Boer goats aged 4–6 months were selected and placed into two treatment groups for 102 days, with water provided ad libitum. During the experiment, six animals died before the collection of samples designated for this metabolomics study due to high parasite burdens. After four weeks into this study, fecal samples were collected for metabolomic analysis.

### 2.1. Sample Collection and Preparation

Fecal samples were collected rectally from each goat naturally infected and put into a labeled Ziplock bag. Samples were kept on ice in a cooler while being transported to the laboratory, and then each fecal sample was split into two separate samples, one for fecal egg count (FEC) and the other for metabolomic analysis. A modified McMaster approach was used to examine individual fecal samples (1–4 g) for FEC, and the results were expressed as eggs per gram (epg). For this study, FEC below 2000 eggs per gram was considered a low parasite burden, and above 2000 eggs per gram was categorized as high infection levels based on similar variation in infection levels among goats used in the study. For proton nuclear magnetic resonance (^1^H-NMR), six samples from each group classified by either extremely high or extremely low fecal egg burden were selected to maximize metabolite discrimination. Twenty-three samples were used for ^1^H-NMR analysis: 12 samples from Bermudagrass fecal matter and 11 from Sunn Hemp. One sample from the Sunn Hemp group was excluded as the processed sample was not enough to carry out data collection. Thirty-eight fecal samples were used for liquid chromatography mass spectometry (LC/MS) analysis and categorized into low or high FEC groups. The fecal samples for metabolomic analysis were stored at −80 °C for both ^1^H-NMR and LC/MS metabolomic analysis.

### 2.2. Feces Extraction for ^1^H-NMR and LC/MS Analysis

Extraction of metabolites was performed according to Jin et al. with some modifications [19]. Specifically, each fecal sample was added to a mortar containing liquid nitrogen and ground with a pestle. The ground fecal samples were aliquoted into 1.5 mL microcentrifuge tubes and kept at −80 °C for both ^1^H-NMR and LC/MS metabolomic analysis.

For each ^1^H-NMR and LC/MS analysis of a fecal sample, one gram of sample was weighed and placed into a 15 mL Eppendorf tube with 10 mL of extraction solution (methanol/Acetonitrile/H_2_O; 2:2:1) and placed in a freezer at −20 °C for 1 h. The samples were then vortexed for one minute, to mix each sample with its extraction solution, followed by ultrasonication of the mixture for 10 min at 40 °C in a sonicating water bath. After sonication, the samples were centrifuged for 15 min at 4 °C at 15,000 rpm, then 600 μL of the upper layer of each sample was collected and placed in a separate 1.5 mL tube. The samples were then vacuum dried overnight to remove the solvent.

### 2.3. ^1^H-NMR Sample Preparation and Data Collection

To each dried sample designated for ^1^H-NMR analysis, 800 μL of deuterium oxide containing the internal standard 0.05% (*w*/*w*) trimethylsilyl propanoic acid (TSP) (Sigma-Aldrich, Saint Louis, MO, USA) was added to reconstitute the dried extract. Then, 600 μL of the aqueous layer of each sample was put into a 5 mm NMR tube for NMR analysis. A Bruker Avance II 400 MHz spectrometer (Bruker Analytik, Rheinstetten, Germany) was used to record proton NMR spectra at a constant temperature of 300 K. The NOESY (Nuclear Overhauser Effect Spectroscopy) pulse sequence, which included 32 scans, 4 dummy scans, and a 2s relaxation delay, was used to obtain the one-dimensional ^1^H-NMR spectra. In addition, 32 K data points and a spectral width of 16 ppm were used to obtain a one-dimensional proton NMR spectrum. Following this, a 0.3 Hz exponential line-broadening was applied to all free induction decays (FIDs). Additionally, every spectrum was manually phased and referenced in accordance with the chemical shift reference TSP at 0.0 ppm using Bruker Analytik’s TopSpin v3.5 software, as previously described by Gines et al. [20].

### 2.4. ^1^H-NMR Data Processing

Chenomx ^1^H-NMR Suite v8.31 software by Chenomx, Incorporated (Edmonton, AB, Canada) was used to identify compounds by comparing information from the literature and accessible databases like the Human Metabolome Database and Madison Metabolomics Consortium Database (Metabolomics Standard Initiative (MSI) Confidence Level: 2). The identified compounds are listed below (Table 1).

Targeted binning was performed using the Mestre Nova (version 14.33; Santiago de Compostela, Spain) NMR processing program. The identified compounds or features were quantified by manually integrating/binning corresponding peaks or clusters, as shown in Table 1. The proton signals from water (4.6–5.0 ppm) as well as the proton signal from the extraction solvent methanol (3.31–3.40 ppm) were excluded from the spectra prior to integration. An Excel file was curated comparing the peak areas of each metabolite/feature for each sample. The dataset was then imported into MetaboAnalyst6.0 for statistical analysis and biomarker identification, normalizing each sample to the TSP peak and utilizing Pareto scaling.

### 2.5. LC/MS Sample Preparation

For LC/MS, all the samples were used, and categorized into low (<2000) and high (>2000) FEC groups to determine discriminatory markers amongst our samples, given that FECs are a measure of genetic-environmental resistance to parasites. The dried samples designated for LC/MS analysis were prepared according to Jin et al. [19]. A 600 μL solution of LC-grade methanol/H_2_O (1:9) was used to reconstitute each sample, followed by vortexing for one minute. Next, samples were ultrasonicated for 10 min at 40 °C followed by centrifugation for 15 min at 4 °C at 15,000 rpm. A total of 600 μL of the supernatant of each sample was then pipetted into a 2 mL screw cap vial bottle for LC/MS analysis.

### 2.6. LC/MS Data Collection and Processing

All LC/MS samples were analyzed using a Vanquish UHPLC system (Thermo Fisher, Waltham, MA, USA) connected to a quadrupole orbitrap mass spectrometer (Orbitrap Exploris 120, Thermo Fisher, Waltham, MA, USA) with electrospray ionization (H-ESI) in both positive and negative modes using Xcalibur software (V4.4.16.14). A 10 μL aliquot of the sample was injected onto a C18 column (ACQUITY UPLC^®^ BEH C18, 1.7 µm, 2.1  ×  50 mm, Waters, Milford, MA, USA) that was kept at 40 °C with a mobile phase flow rate of 200 μL/min using solution A (99.9% water with 0.1% formic acid) and solution B (95% acetonitrile 5% and water with 0.1% formic acid).

After maintaining the gradient at 0% B for 1.5 min, the run increased linearly to 40% B at 3 min, reaching 100% B at 12 min. The total analysis time was 18 min, with one minute at 0% B. The flow was diverted to waste during the first minute of analysis and again after 15 min. The MS operated in small molecule mode, with a scan range of 100–1000 *m*/*z*, a resolution of 60,000, a standard automatic gain control (AGC) target, radio frequency (RF) lens set to 70%, auto maximum injection duration, and EASY-IC (internal calibration) activated at the start of the run.

The spray voltage was set to 3300 V for positive mode and 2300 V for negative mode. The ion transfer tube temperature was maintained at 320 °C, while the vaporizer temperature was adjusted to 275 °C. The data-dependent collection threshold was set at 20,000, with a window size of 1.7 Da. Normalized Higher-Energy Collisional Dissociation (HCD) collision energy was applied at 15%, 30%, and 75%, using a typical resolution of 15,000, and the AGC goal was set to the standard for the four dependent scans. Blank injections were used to create a targeted mass exclusion list during analysis. Compound Discoverer software v3.2 facilitated compound identification and peak area measurement.

## 3. Results

### 3.1. Bermudagrass and Sunn Hemp FEC Measurements

**Figure 1:**
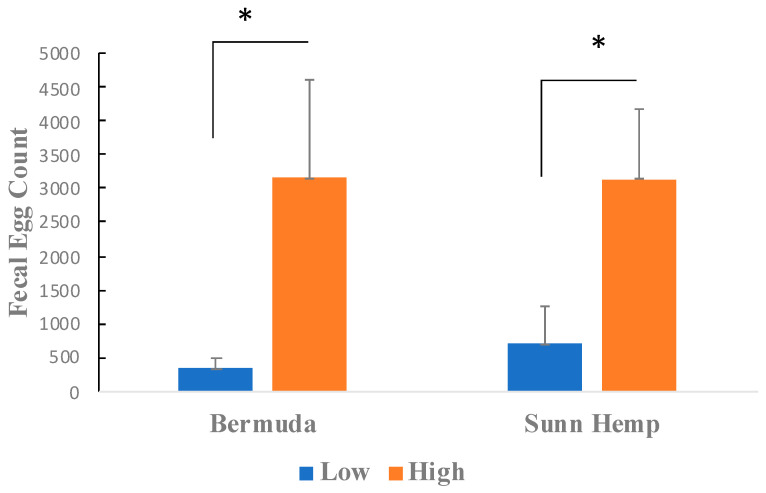
Comparison of Haemonchus contortus egg burden in feces for goats categorized into low and high total fecal egg count groups after 4 weeks of grazing on Bermudagrass or Sunn Hemp paddocks. Asterisks indicate statistical significance (*p* < 0.05). Standard deviations are presented.

The bar graph illustrated in Figure 1 compares the effects of two forages (Bermudagrass and Sunn Hemp) on two groups of goats (low and high parasite loads) by showing their total fecal egg count (FEC) after 4 weeks of dietary intervention. Significant variations in parasite load were seen within each forage type when comparing the low and high groups.

### 3.2. Metabolomic Profile for Goat Feces

**Figure 2:**
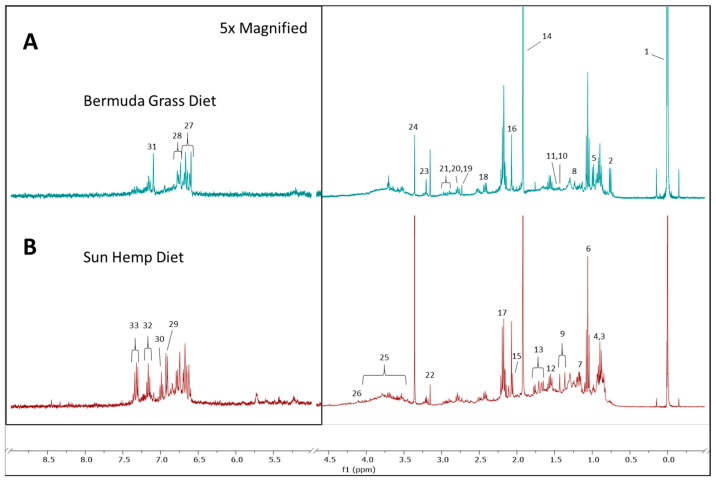
Representative ^1^H NMR spectra of fecal metabolomes of goats grazing on Bermuda grass field (**A**) or a Sunn Hemp paddock (**B**). 1: 3-(trimethylsilyl)propionic acid (TSP); 2: Unknown metabolite; 3: Butyrate acid/FAs 4: Pantothenic acid; 5: valine; 6: propionate; 7: ethanol; 8: Unknown metabolite; 9: Lactate; 10: Unknown metabolite; 11: Alanine; 12: Butyrate; 13: Arginine/Lysine/Unknown; 14: Acetic acid; 15: N-acetyllysine/Acetylated proteins; 16: N-acetyl/ Proteins/Carbohydrates; 17: Propionate/Butyrate; 18: Succinate/unknown metabolite; 19: Unknown metabolite; 20: Unknown; 21: Unknown metabolite; 22: Dimethyl sulfone; 23: Choline, Phosphocholine, Carnitine; 24: Methanol (excluded); 25: Glucose/Sugar; 26: Lactate; 27: Unknown metabolite; 28: Unknown metabolite; 29: Tyrosine; 30: Unknown metabolite; 31: Unknown metabolite; 32: Unknown metabolite; 33: Phenylalanine.

Metabolomic profiles of the goat fecal samples were constructed using ^1^H-NMR spectroscopy. The analysis of the metabolomic profiles included thirty-two metabolic features. Different metabolites were found among the samples, and spectral comparisons showed Sunn Hemp had a greater diversity of metabolites than Bermudagrass. Among the metabolites identified, were some organic acid metabolites (butyrate acid, propionate, acetic acid, and succinate), sugar (glucose), amino acids (valine, alanine, arginine, tyrosine, and phenylalanine), vitamin (pantothenic acid), and other metabolites (n-acetyllysine, acetylated proteins, dimethyl sulfone, choline, phosphocholine, carnitine, and lactate). We also included some unknown metabolite signals to increase potential biomarker identification (Figure 2).

**Figure 3:**
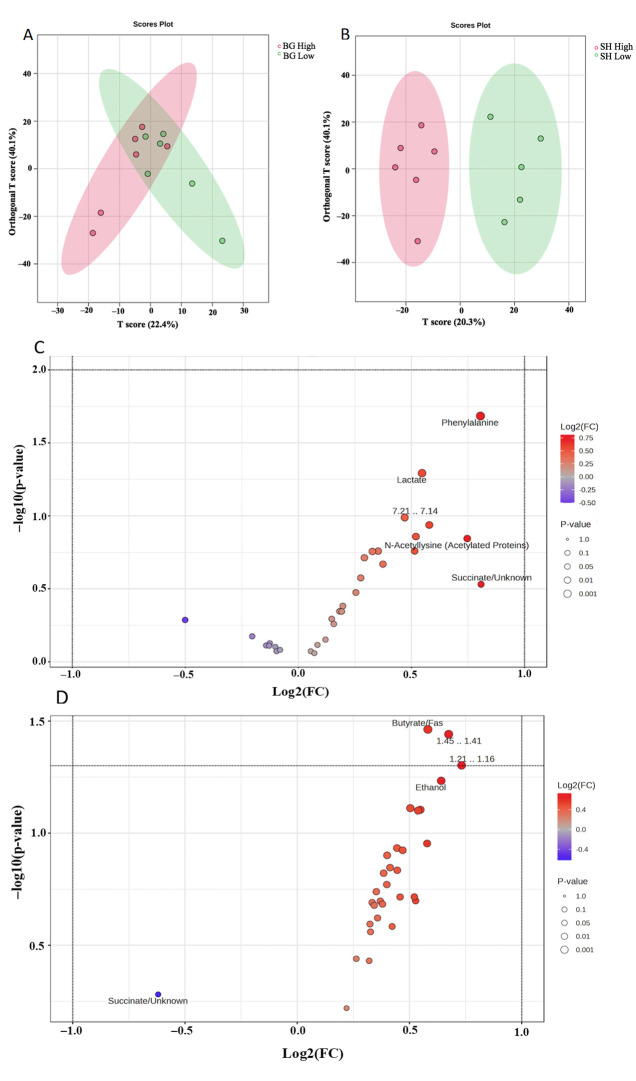
Orthogonal partial least squares discrimination analysis of high (>2000; red) vs. low (<2000; green) fecal egg counts of goats on Bermudagrass diet (**A**) or Sunn Hemp diet (**B**) using NMR data. One outlier sample from the Sunn Hemp diet was excluded from analysis. Volcano plots of goat fecal differential metabolites between Bermudagrass diet (**C**) or Sunn Hemp diet (**D**) using NMR data; n = 6 samples per group. Red dots indicate upregulation, and blue dots represent downregulated metabolites in goats with high parasite burden.

Figure 3 illustrates the comparison of metabolomic data generated from the ^1^H NMR spectra of fecal samples from low- and heavy parasite burden goats fed Bermudagrass and Sunn Hemp using orthogonal partial least square dicriminatory analysis (OPLS-DA). Principal component analysis (PCA) and partial least square dicriminatory analysis (PLS-DA) showed little separation (Supplementary Figures S1 and S2); thus, the OPLS, which showed the most separation between groups, was considered for further statistical analysis. The OPLS-DA score plot along the T-score for component 1 for Bermuda and Sunn Hemp represented 22.4% (Figure 3A) and 20.3% (Figure 3B) of the total variation, respectively. An OPLS-DA score plot for Bermuda and Sunn Hemp was used to evaluate different performances among both, and a permutation test (set permutation number 20) was used to verify the model. In the feces for Sunn Hemp, R^2^Y = 0.383 and Q^2^ = −0.878. The *p*-values of permutation for feces were 0.9 (R^2^Y) and 0.85 (Q^2^) for Sunn Hemp. While for Bermuda R^2^Y = 0.399 and Q^2^ = 0.0595, and the *p*-values were 0.75 for R^2^Y and 0.4 for Q^2^. The R^2^Y and Q^2^ values being low, 0.4, demonstrate that the model lacked stability and consistency for the feces samples for Bermuda and Sunn Hemp using NMR. The feces samples of Bermudagrass and Sunn Hemp groups contained 31 metabolites that were not significantly affected by parasite burden, while 2 metabolites were found to be significantly altered (as shown in Figure 3C,D). The distribution and fold changes in these metabolites were displayed by creating a volcano plot.

#### 3.2.1. Variable Importance Plot (VIP)

**Figure 4:**
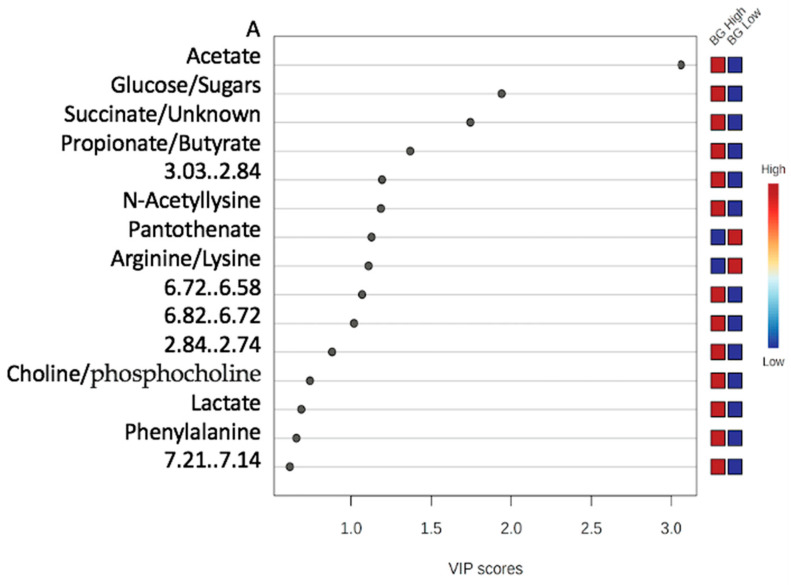

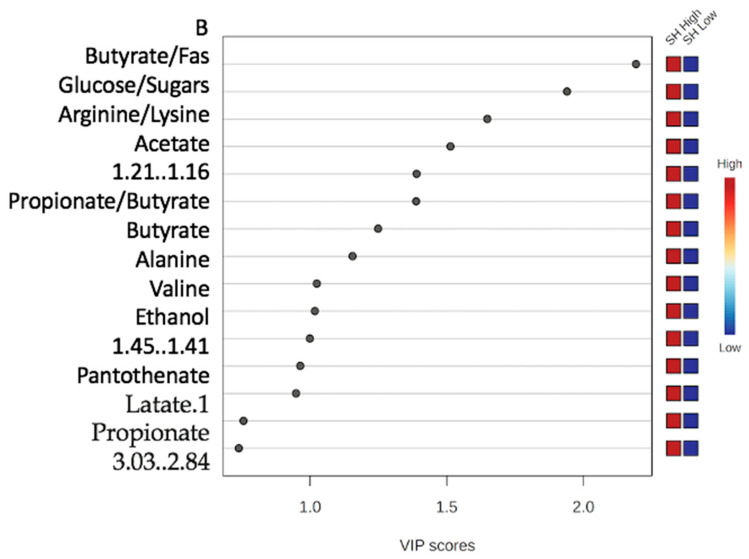
Variable importance plot ((**A**), Bermudagrass), ((**B**), Sunn Hemp) to analyze the metabolic composition of fecal samples from goats with high (>2000) or low (<2000) fecal egg counts while grazing on Sunn Hemp or Bermudagrass paddock. Each group consisted of twelve samples. Metabolites that are more abundant between groups are displayed in red on the heat map to the right, while those that are less abundant are displayed in blue.

VIP score plots comprising 15 metabolites/features for each group were generated by multivariate statistical analysis, with those having a score greater than one (Figure 4). As a result, 15 metabolites/features for Sunn Hemp and 15 metabolites/features for Bermudagrass contributed significantly (VIP > 1) to the OPLS-DA classification of the major feature groups. Among the 15 metabolites for Bermudagrass, we identified one aromatic amino acid (phenylalanine), two organic acids (lactate, acetate), one vitamin (pantothenate), one basic amino acid (arginine), one fatty acid (propionate/butyrate), one tricarboxylic acid cycle intermediate (succinate), one acetylated amino acid (n-acetyllysine), one phospholipid precursor/alcohol (choline/phosphocholine), one monosaccharide/carbohydrate (glucose/sugars), and five unknown features ranging from 7.21 to 7.14, 2.84 to 2.74, 6.82 to 6.72, 6.72 to 6.58, and from 3.03 to 2.84 ppm. Among the top discriminatory features shown for Sunn Hemp were two short chain fatty acids (butyrate, propionate), four amino acids (alanine, valine, arginine/lysine), one alcohol (ethanol), two organic acids (lactate, acetate), one vitamin B5 (pantothenate), monosaccharide/carbohydrate signals (glucose/sugars), and three unknown metabolites ranging between 1.45 and 1.41, 1.21 and 1.16, and between 3.03 and 2.84 ppm for Sunn Hemp. The metabolite driving the greatest separation in Figure 4 was acetate for Bermudagrass, whereas butyrate was the main contributor for Sunn Hemp (Figure 4).

#### 3.2.2. Pathway Analysis for ^1^H-NMR

**Figure 5:**
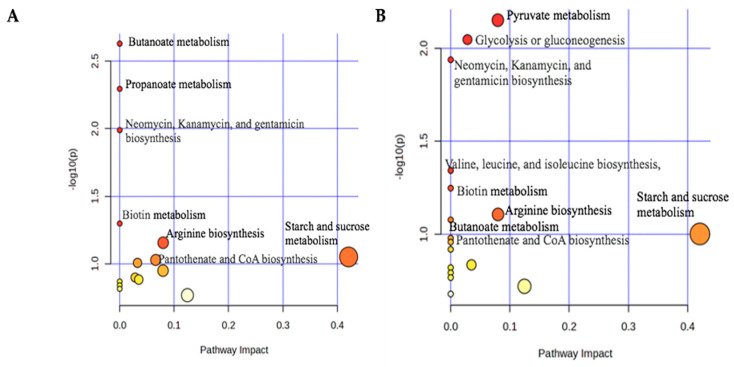
MetaboAnalyst6.0 was used to do the analysis, which was based on the Capra hicus KEGG Pathway analysis summary plots using goat feces from Bermudagrass (**A**) and Sunn Hemp (**B**) groups. Both plots show the top 15 pathways for Bermudagrass and the top 17 pathways for Sunn Hemp.

A pathway analysis was performed using the top discriminatory metabolites for goats fed Bermudagrass and Sunn Hemp to connect the identified metabolites with their respective metabolic pathways. Figure 5 displays the metabolic pathways implicated using the reference Capra hircus (goat) KEGG (Kyoto Encyclopedia of Genes and Genomes) database. There were similar pathways for both diets, such as butanoate metabolism, propanoate metabolism, neomycin, kanamycin and gentamicin biosynthesis, biotin metabolism, arginine biosynthesis, starch and sucrose metabolism, pantothenate and Coenzyme A (CoA) biosynthesis, pyruvate metabolism, glycolysis or gluconeogenesis, galactose metabolism, alanine, aspartate and glutamate metabolism, lysine degradation, glyoxylate and dicarboxylate metabolism, arginine and proline metabolism. We found that the citrate cycle was present in Bermudagrass fed goats, while valine, leucine, isoleucine biosynthesis, and seleno compound metabolism were present in Sunn Hemp fed goats. According to the examination of fecal metabolites, the primary pathway associated with parasite reduction was butanoate (butyrate) metabolism, propanoate (propionate) metabolism, arginine biosynthesis, arginine and proline metabolism. Among the pathway analyses, some of the metabolite pathways were significantly associated with both forages, like neomycin, kanamycin, and gentamicin biosynthesis, biotin metabolism, while valine, leucine, and isoleucine biosynthesis were significant only in Sunn Hemp at *p* < 0.05 (Figure 5).

#### 3.2.3. Fecal Metabolomics Profile for LC/MS

**Figure 6:**
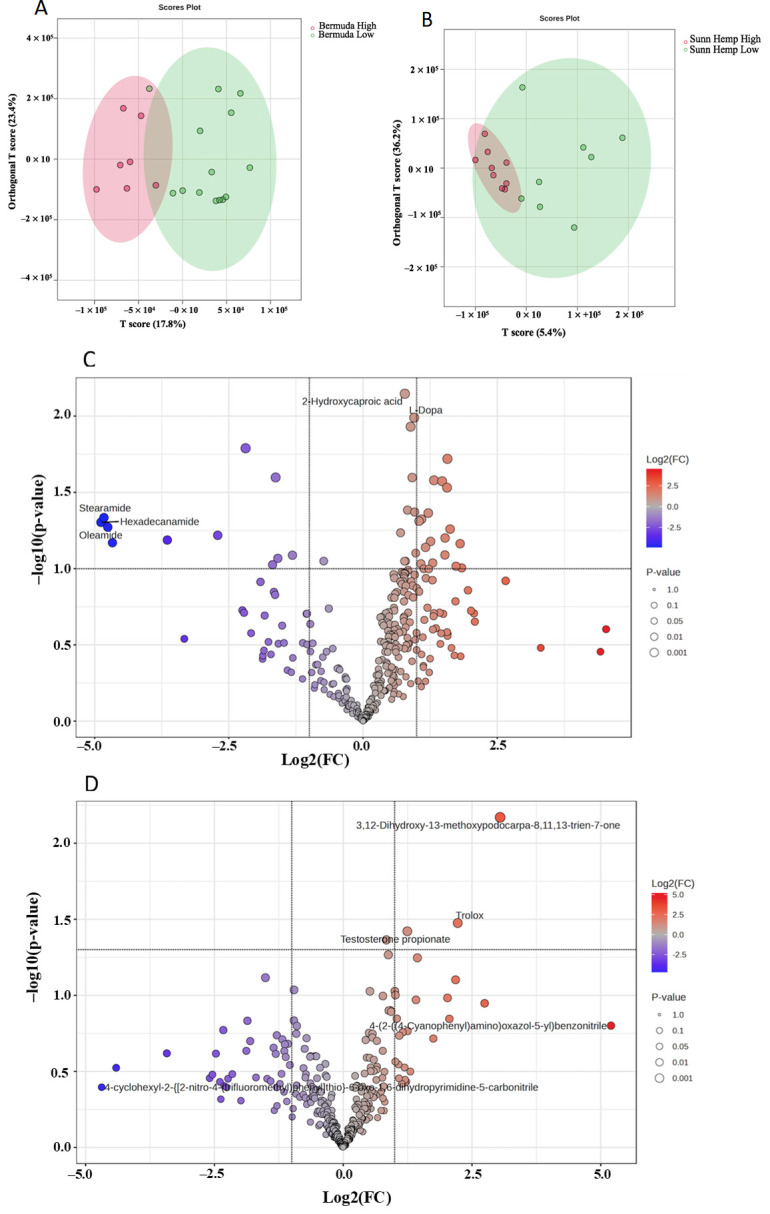
OPLS-DA score plots: orthogonal partial least squares discrimination analysis of high (red) vs. low (green) fecal egg counts of goats on Bermudagrass diet (**A**) or Sunn Hemp diet (**B**) using LC/MS data. Volcano plot of goat fecal differential metabolites between Bermudagrass (**C**) and Sunn Hemp (**D**). Red dots indicate upregulation, and blue dots represent downregulated metabolites in goats with high parasite burden.

The score plots shown in Figure 6A,B detail the difference in the metabolite profiles between Bermuda and Sunn Hemp goat fecal samples, as evidenced by the orthogonal partial least squares discriminant analysis (OPLS-DA) score plots along T-score component 1 for Bermudagrass and for Sunn Hemp, represented 17.8% (Figure 6A) and 5.4% (Figure 6B) of the total variation). PCA and PLS-DA showed little separation (Supplementary Figures S3 and S4); thus, the OPLS, which showed the most separation between groups, was considered for further statistical analysis. The feces samples of Bermudagrass groups contained 317 metabolites, of which 7 were significantly upregulated, 4 were significantly downregulated (*p* < 0.05 and FC > 2). Sunn Hemp groups contained 317 metabolites that were affected by parasite burden, while 3 metabolites were found to be significantly altered (as shown in Figure 6C,D). The distribution and fold changes in these changes in these metabolites were displayed by creating a volcano plot (Figure 6C,D). An OPLS-DA score plot for Bermuda and Sunn Hemp was used to evaluate different performances among both, and permutation tests (set permutation number 100) were used to verify the model. In the feces for Sunn Hemp, R^2^Y = 0.798 and Q^2^ = −0.137. The *p*-values of permutation for feces were 0.21 (R^2^Y) and 0.36 (Q^2^) for Sunn Hemp. While for Bermuda R^2^Y = 0.965 and Q^2^ = −0.457, and the *p*-values were 0.04 for R^2^Y and 0.7 for Q^2^. The R^2^Y and Q^2^ values being low, 0.4, demonstrate that the model lacked stability and consistency for the feces samples for Bermuda and Sunn Hemp using LC/MS. Additionally, a Q^2^ value well below 1 showed that the OPLS-DA model lacked high predictability.

#### 3.2.4. Variable Importance Plot (VIP) and Metabolites

**Figure 7:**
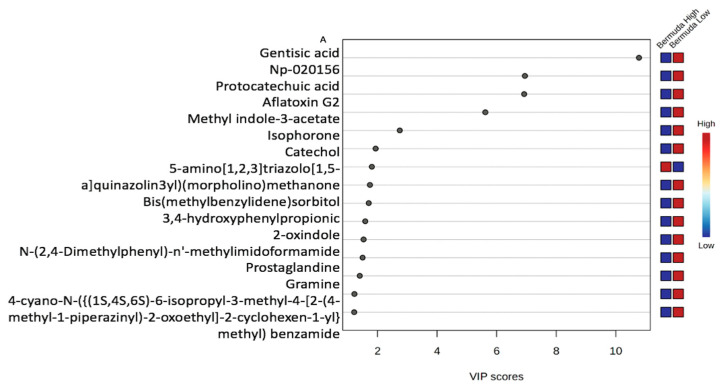

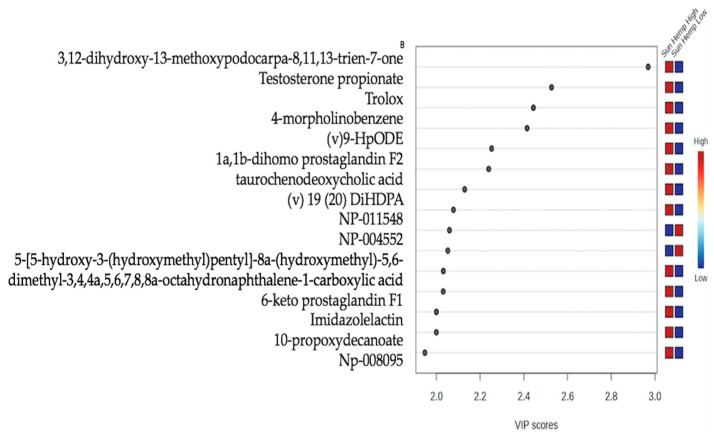
The VIP ((**A**) Bermudagrass and (**B**) Sunn Hemp) illustrates the top discriminatory metabolites of fecal samples from goats with high (>2000) or low (<2000) fecal egg counts while grazing on Sunn Hemp or Bermudagrass paddocks. Each group consisted of twelve samples. One outlier sample from the Sunn Hemp diet’s low parasite load group was excluded from the analysis.

A variable importance plot (VIP) comprising 15 metabolites for each group was generated by multivariate statistical analysis of all metabolites, as shown in Figure 7. The 15 metabolites in the VIP for Bermudagrass include gentisic acid; np-020156; protocatechuic acid; aflatoxin G2; methyl indole-3-acetate; isophorone; catechol; 5-amino[1,2,3]triazolo[1,5-a]quinazolin3yl)(morpholino)methanone; 3,4-hydroxyphenylpropionic; 2-oxindole; n-(2,4-Dimethylphenyl)-n’-methylimidoformamide; prostaglandine; gramine; 4-cyano-N-({(1S,4S,6S)-6-isopropyl-3-methyl-4-[2-(4-methyl-1-piperazinyl)-2-oxoethyl]-2-cyclohexen-1-yl} methyl) benzamide; and bis(methylbenzylidene)sorbitol. The metabolite driving the greatest separation in Figure 7 for Bermudagrass was gentisic acid. Three other metabolites, np-020156 (unknown), protocatechuic acid, and aflatoxin G2), also contributed significantly to the separation.

The 15 metabolites in the VIP for Sunn Hemp were 3,12-dihydroxy-13-methoxypodocarpa-8,11,13-trien-7-one; testosterone propionate; trolox; 4-morpholinobenzene; (v)9-HpODE; 1a,1b-dihomo prostaglandin F2; taurochenodeoxycholic acid; (v) 19 (20) DiHDPA; NP-011548; NP-004552; 5-[5-hydroxy-3-(hydroxymethyl)pentyl]-8a-(hydroxymethyl)-5,6-dimethyl-3,4,4a,5,6,7,8,8a-octahydronaphthalene-1-carboxylic acid; 6-keto prostaglandin F1; imidazolelactin; 10-propoxydecanoate; and np-008095. The metabolite driving the greatest separation in Figure 7 for Sunn Hemp was 3,12-dihydroxy-13-methoxypodocarpa-8,11,13-trien-7-one. The next three metabolites that contributed to the separation were testosterone propionate, Trolox, and 4-morpholinobenzene (Figure 7).

#### 3.2.5. Pathway Analysis for LC-MS

**Figure 8:**
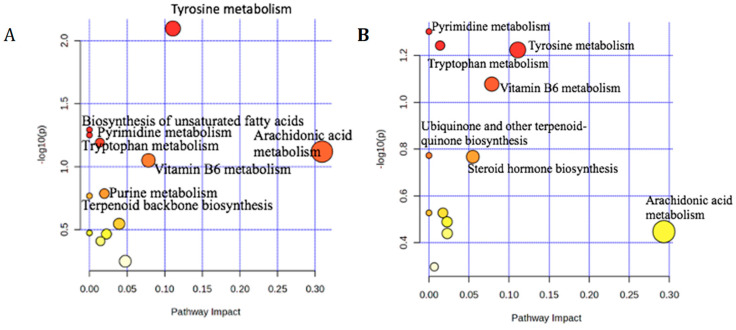
Pathway analysis summary plots using goat feces from Bermudagrass (**A**) and Sunn Hemp (**B**). Both plots show the top 14 pathways for Bermudagrass and the top 12 pathways for Sunn Hemp implicated in *H. contortus* infection.

The most discriminatory metabolites (VIP > 1) found in the goat fecal samples were used in pathway analysis, which connected these metabolites to the corresponding metabolic processes. The top discriminatory metabolites from each forage group were linked to their relevant pathways (shown in Figure 8) using the KEGG database for Capra hircus (goat). There were several similar pathways for both feces analyses, such as tyrosine metabolism, biosynthesis of unsaturated fatty acids, pyrimidine metabolism, tryptophan metabolism, arachidonic acid metabolism, vitamin B6 metabolism, purine metabolism, terpenoid backbone biosynthesis, glycosylphosphatidylinositol (GPI)-anchor biosynthesis, fatty acid elongation, fatty acid degradation, valine, leucine and isoleucine degradation, fatty acid biosynthesis, and steroid hormone biosynthesis. This finding includes some lipid and some amino acid metabolism for both forages (Figure 8).

## 4. Discussion

This study investigated the metabolite composition of feces from Spanish Boer goats grazing on either Bermudagrass or Sunn Hemp paddocks analyzed using ^1^H- NMR and LC/MS. There are few metabolomic studies on goat feces, and as far as we know, no study has looked at how *H. contortus* affects goat metabolite profiles under different diets. In this study, we used ^1^H- NMR, and LC/MS to identify and quantify the metabolites in the feces, and chemometric methods such as orthogonal partial least square dicriminatory analysis (OPLS-DA), volcano plot, variable importance plot (VIP), and pathway analysis to identify potential biomarkers in feces of goats on a diet of Bermudagrass or Sunn Hemp with varying levels of *H. contotus* infection. We have identified putative biomarkers for parasite load and for resistance to parasite infection.

The volcano plot showed no significant metabolites from Bermudagrass or Sunn Hemp on the ^1^H- NMR. Meanwhile, LC/MS from Bermudagrass revealed significant upregulated metabolites such as methyl indole-3-acetate; np-020156; np019748; gramine; 2,2,6,6-tetramethyl-1-piperidinol (TEMPO); 3-tertbuthyladipic acid, and 5-Hydroxyindole-3-acetic acid; and significantly downregulated metabolites known as hexadecanamide; stearamide; aflatoxin G2; and palmitic acid in goats with higher levels of *H. contortus* infection. The Sunn Hemp diet indicated 3,12-dihydroxy-13-methoxypodocarpa-8,11,13-trien-7-one; trolox, and testosterone propionate as significantly upregulated metabolites for goats with higher levels of infection.

Moreover, from VIPs, we observed a wide range of metabolites from goats that grazed Bermudagrass and Sunn Hemp, revealing several significant metabolic pathways that may be connected to the host parasite, microbial interactions, and energy metabolism. In total, 10 discriminatory metabolites (VIP > 1) were revealed for ^1^H-NMR, while LC/MS analysis revealed a total of 115 (Bermudagrass) and 113 (Sunn Hemp). Both techniques were used in our study to detect the most sensitive metabolites and great characterization from low and high egg counts from both forages. Similarly, Martias et al. reported that using different techniques allowed the wide metabolite coverage and maximum reproducibility in the extraction procedure [10].

The different botanical and nutritional compositions of Sunn Hemp and Bermudagrass are largely responsible for the difference in their metabolite profiles. Sunn Hemp is a legume that is high in protein, soluble carbohydrates, and secondary metabolites, whereas Bermudagrass is a grass that is mostly made up of structural carbohydrates. Sunn Hemp contains secondary metabolites that may affect rumen microbial activity by blocking specific microorganisms and changing fermentation pathways, changing the final metabolite balance. Consequently, Sunn Hemp exhibits increased soluble nutrient availability and microbial turnover, as indicated by the presence of acetate, glucose/sugars, succinate (an intermediary associated with propionate synthesis), propionate, and butyrate. Glucose and sugar suggest greater availability of soluble carbohydrates, and acetate is a major product of structural carbohydrate breakdown. Propionate and butyrate together produce energy-rich volatile fatty acids that aid in the host’s gluconeogenesis, while succinate, an intermediary in the propionate and butyrate pathways, indicates active microbial turnover.

Bermudagrass, on the other hand, exhibits top metabolites such as propionate, acetate, glucose, arginine, and lysine from amino acid metabolism, butyrate, and fatty acids from lipid-associated metabolism and fiber fermentation. Overall, the top discriminatory metabolites show that Bermudagrass favors fiber-driven butyrate production and amino acid catabolism, while Sunn Hemp supports pathways linked to sugar utilization and fermentation intermediates. This highlights how forage type influences rumen metabolism through nutrient content and secondary compound effects.

A study carried out by Malheiros et al. [21], showed that acetates, propionates, and butyrate were the main abundant metabolites found in samples of feces and in ruminal fluid of Nelore steers. Similarly, Martias et al. used ^1^H- NMR and UHPLC-HRMS and reported the presence of these same metabolites in goat milk, feces, and plasma [10]. These metabolites (acetate, propionate, and butyrate) were found in fecal samples from our work with parasite-infected goats, indicating that these metabolites are consistently prominent throughout ruminant species regardless of physiological condition, and differential expression of these metabolites may indicate severity of infection. This data emphasizes how goats and cattle have similar metabolic profiles, highlighting the significance of these metabolites for ruminant gut health and function. In addition, our findings had a similar metabolomic profile compared to the feces metabolome of clinical mastitis cows reported by Zhu et al. [18]. This similarity emphasizes how these metabolites may serve as cross-species universal indicators of host pathogen interactions. These metabolic markers could serve as potential biomarkers for diagnostic tools, tracking the course of diseases, and discovering treatment targets that can enhance animal health and production in dairy and small ruminant systems. In contrast, Liu et al. [22] found, through enrichment pathways analysis (tryptophan, vitamin B6, and steroid hormone metabolism), that a lower-protein diet changes the goat fecal microbiome. Our study also indicated changes in fecal metabolites and pathways involving similar metabolites, highlighting similar metabolic adaptations. All of these results indicated that metabolic changes in the common biomarkers of gut health in goats can be caused by parasite infection and diet.

Samples associated with high fecal egg counts contained significantly higher levels of these metabolites. Among them, the first was 3,12-dihydroxy-13-methoxypodocarpa-8,11,13-trien-7-one, commonly called nimbionol (a diterpenoid plant secondary metabolite), is known to have antimicrobial properties and may contribute to Sunn Hemp’s bioactive chemistry that influences gut microbial composition [23]. The second, testosterone propionate, an androgenic steroid, was also detected and may indirectly modulate gut microbial communities through hormonal signaling [24]. The third top discriminatory metabolite, trolox, is a derivative of vitamin E that is used as a standard for determining the antioxidant content in food [25]. Considering that vitamin E derivatives are frequently found in Sunn Hemp (seeds and flowers), it might be possible that trolox comes from plant sources [26]. Further studies are needed to elucidate the impact of trolox in the goat’s microbiome and why its concentrations were higher in Sunn Hemp fed goats with high parasite burden. Another top discriminatory metabolite, 4-morpholinobenzene, might probably be found in the feces due to microbial activity in goat gastrointestinal gut and converted into a variety of metabolites. Further studies are needed to validate this compound. Collectively, these metabolites underscore the role of Sunn Hemp-derived compounds and host responses in shaping possible parasite infection dynamics in goats. Elevated discriminatory metabolites in high-parasite samples may reflect parasite-driven oxidative stress, immune responses, or shifts in microbial metabolism. Statistically, they were significantly different between high and low parasite groups, highlighting their potential as biomarkers of infection. Their consistent differential abundance supports their possible benefit in monitoring parasite burden in ruminants.

Small ruminant fecal samples commonly contain gentisic acid, a plant-derived phenolic compound with anti-inflammatory and antioxidant properties that is mostly thought to be a byproduct of microbial metabolism and forage digestion [27]. Ingestion of infected foods and feedstuffs is the principal way that aflatoxins enter the mammalian system, and the alimentary tract is the predominant site of absorption. Our study found the presence of aflatoxin G2 in goat feces fed different forages, which agrees with the study performed by Tsiplakou et al. [28]. Generally, aflatoxin contamination occurs in different livestock that consume maize, and other feeds by specific aspergillus flavus fungal species [29]. Similarly, another study reported the presence of aflatoxin G2 in animals fed by [30]. Puga-Torres, Ron & Gomez reported that aflatoxin exposure occurs when contaminated or moldy feed is consumed [31]. The ingested poison in ruminants is partially broken down by the rumen before being eliminated from the body through secretions such as milk and urine [31]. Small ruminant feces contain protocatechuic acid, a phenolic metabolite obtained from plants that is created when microorganisms break down dietary polyphenols. It has anti-inflammatory, gut-health-promoting, and antioxidant properties; its presence reflects microbial activity and pasture composition [32]. The unknown chemical NP-020156 was found in high-FEC samples, which may indicate a connection to parasite stress. These results show the intricate relationship between parasitic infections and plant metabolites in ruminants, underscoring the necessity of thorough research to clarify their functions and potential as parasite burden biomarkers.

The secondary metabolites in Sunn Hemp can have a significant impact on microbiome fermentation and host metabolism. The microbiome populations, promoting or inhibiting particular metabolic pathways, can change the synthesis of downstream metabolites such as volatile fatty acids. The two forages’ nutritional and bioactive chemical content is reflected in the differences in their metabolites and parasite burdens. Bermudagrass fiber drives fermentation and the metabolism of phenolic acids. Sunn Hemp encourages the use of soluble carbohydrates, antioxidant activity, and the synthesis of metabolites that are high in energy. By influencing host energy metabolism, antioxidant capacity, and resistance to oxidative stress, these top discriminatory metabolites reveal functional differences that affect animal health, growth, and productivity.

The indication of neomycin and kanamycin metabolites’ function in the pathway analysis could alter immunological responses and reduce susceptibility to gastrointestinal parasites like Haemonchus contortus. Furthermore, the identification of kanamycin and neomycin biosynthesis pathways in our analysis supports the idea that the microbiota that lives in the gut has a major role in the production of antimicrobial substances. The notion that microbial-derived aminoglycosides may aid in suppressing or inhibiting parasite establishment is supported by Kim et al. [33], who found that gut microorganisms with neomycin resistance protected against toxic exposure and disturbed worm development.

Data from our results can shed more light on how diet affects parasite burdens. Our results demonstrate the presence of lipid metabolism, including the biosynthesis of unsaturated fatty acids and the generation of fatty acids in goat feces. Fatty acids, particularly unsaturated ones, may alter the integrity of the gut membrane, host immune responses, and microbial populations, which can have an impact on parasite loads by reducing the conditions that are conducive to parasite life. According to Liu et al. [22], diet can change the metabolome of goat feces through pathways such as the metabolism of fatty acids, tryptophan, and vitamin B6. Our findings demonstrate that nutrition and parasite infection influence the same metabolic pathways. Therefore, these metabolites could be used as novel biomarkers to detect gastrointestinal parasite burden in goats.

Moreover, other pathways like steroid biosynthesis, unsaturated fatty acid biosynthesis, arachidonic acid metabolism, amino acid metabolism (tyrosine, and phenylalanine) were present in our study as metabolisms affected by parasite burden. Similar pathways were identified in a study by Zhu et al. [18] in dairy cows suffering from subclinical mastitis. This could be a reflection of the host immune system’s reaction to the presence of the different pathogen [34]. These pathways were present in our findings, which may reflect the same response due to parasitic infections. These changes show that fatty acids are important indicators of host–parasite interaction in addition to being essential for energy and cell structure. As consistently reported across ruminant species and health conditions, our study shows that parasite infection and forage diet in goats change important metabolic pathways, especially those involving short-chain fatty acids, lipid metabolism, amino acid metabolism (tryptophan, tyrosine, and phenylalanine), and vitamin B6.

## 5. Conclusions

Our understanding of natural parasite control mechanisms in small ruminants highlights potential discriminatory metabolites and pathways linked with parasite burden. The development of sustainable parasite management strategies is based on the critical role of short-chain fatty acid metabolism, specifically the production of butyrate and propionate, amino acids (alanine, valine), and lipids (fatty acids/FAs). In sustainable ruminant production systems, the varying impacts of Sunn Hemp and Bermudagrass on fecal metabolite profiles may show how dietary manipulation might improve natural parasite resistance, providing prospective substitutes to identify novel biomarkers.

To our knowledge, this is one of the few studies that used untargeted ^1^H-NMR and LC/MS metabolomic analysis to provide quantitative data about the goat feces metabolome. To elucidate the processes of goat metabolome variation influenced by diet and parasite loads, a number of analyses and top discriminatory metabolites were observed, which could be possible biomarkers. Our results offer insight into the metabolic processes of goat feces, which is helpful in the development of controlled ways to improve the goat’s health and promote sustainable parasite control in small ruminants.

## Figures and Tables

**Table 1:** List of compounds identified in the ^1^H-NMR spectra and chemical shift ranges in parts per million (ppm) used for quantification.

Metabolite	Range (ppm)
TSP	−0.09–0.1
Unknown	0.68–0.8
Butyrate acid/FA signals	0.81–0.93
Panthotenic acid	0.93–0.96
Valine	0.97–1.01
Propionate	1.07–1.08
Unknown	1.13–1.16
Ethanol	1.16–1.21
Lactate	1.32–1.36
Unknown	1.41–1.45
Alanine	1.44–1.5
Butyrate	1.52–1.6
Arginine/Lysine/Unknown	1.61–1.85
Acetic Acid	1.86–1.94
N-acetyllysine acetylated proteins	1.98–2.02
N-acetyl/proteins/Carbohydrates	2.04–2.09
Propionate/Butyrate	2.13–2.24
Succinate/Unknown	2.38–2.45
Unknown	2.71–2.74
Unknown	2.74–2.84
Unknown	2.84–3.04
Dimethyl sulfone	3.14–3.17
Choline, phosphocholine/carnitine	3.17–3.26
Glucose/sugar	3.4–3.9
Lactate	4.07–4.17
Unknown	6.58–6.72
Unknown	6.72–6.82
Tyrosine	6.89–6.96
Unknown	7.07–7.14
Unknown	6.96–7.05
Unknown	7.14–7.21
Phenylalanine	7.28–7.39

## Data Availability

The original contributions presented in this study are included in the article/supplementary material. Further inquiries can be directed to the corresponding author.

## Supplementary Materials

The following supporting information can be downloaded at: https://www.mdpi.com/article/10.3390/metabo15110741/s1, Figure S1: PCA Scores Plot from NMR analysis; Figure S2: PLSDA Scores Plot from NMR analysis; Figure S3: PCA Scores Plot from LC/MS analysis; Figure S4: PLSDA Scores Plot from LC/MS analysis.
