# Supplementary material for: Comparison of Metabolomic Signatures Between Low and Heavy Parasite Burden of Haemonchus contortus in Meat Goats Fed with Cynodon dactylon (Bermudagrass) and Crotalaria juncea L. (Sunn Hemp)
Source: Metabolites. 2025 Nov 14;15(11):741. doi: 10.3390/metabo15110741 (PMC12654777; doi:10.3390/metabo15110741)

Supplementary Data for metabolomic of goat's feces from Bermudagrass and Sunn Hemp using ^{1}H-NMR and LC/MS methods

Figure S1. PCA Scores Plot from NMR analysis for Bermudagrass group (left) and Sunn Hemp group (right)

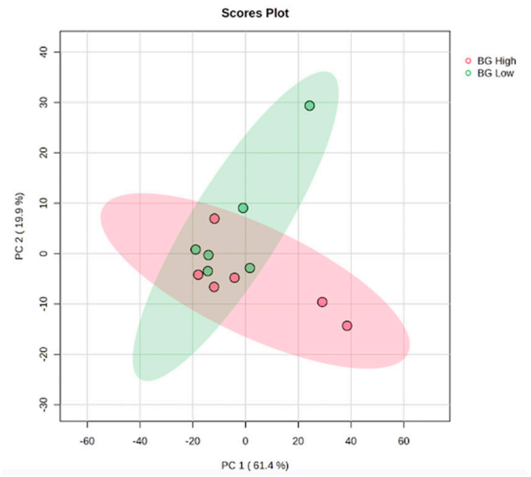

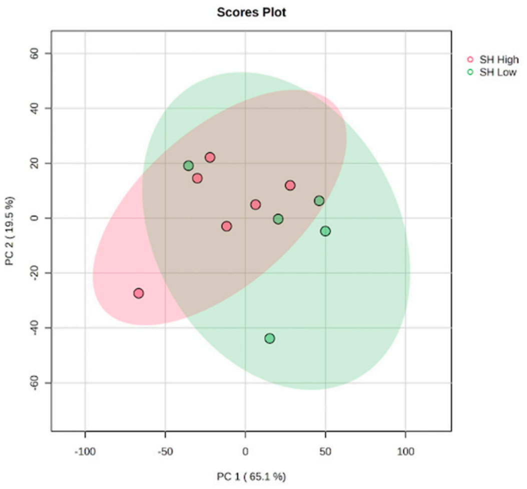

Figure S2. PLSDA Scores Plot from NMR analysis for Bermudagrass group (left) and Sunn Hemp group (right)

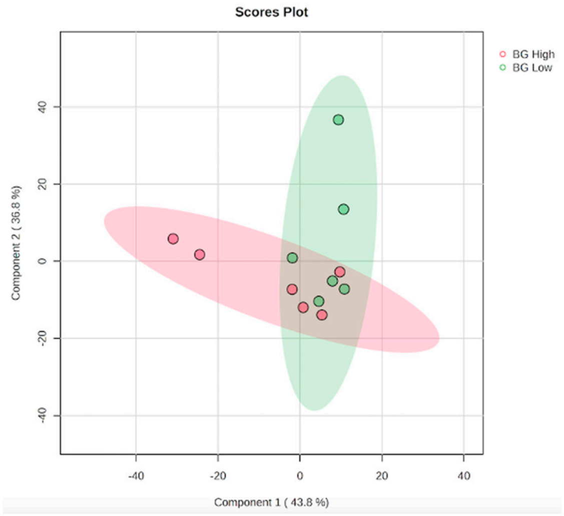

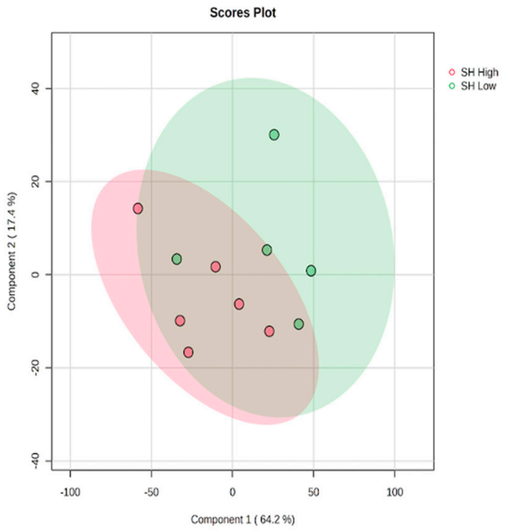

Figure S3. PCA Scores Plot from LC/MS analysis for Bermudagrass group (left) and Sunn Hemp group (right)

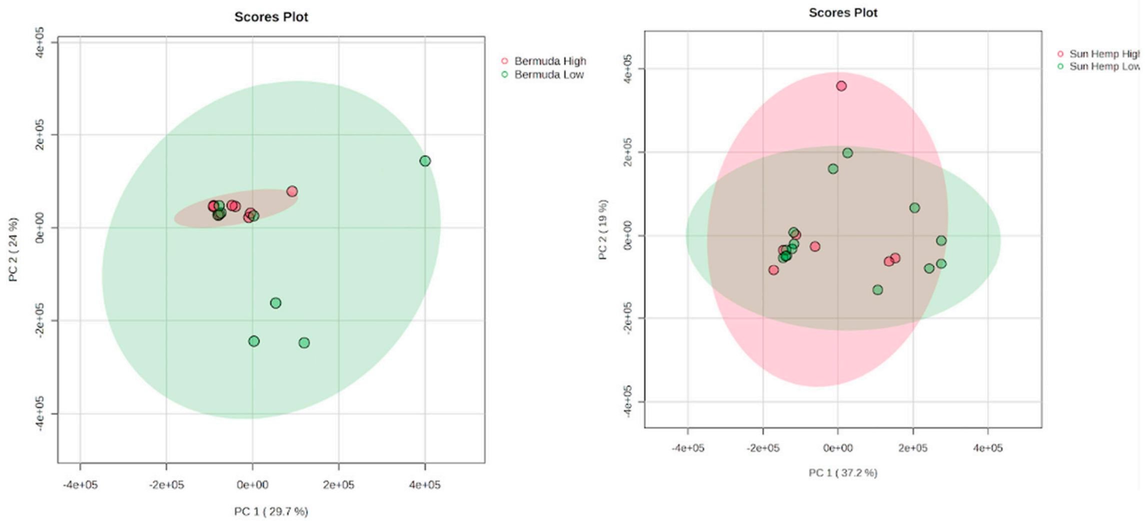

Figure S4. PLSDA Scores Plot from LC/MS analysis for Bermudagrass group (left) and Sunn Hemp group (right)

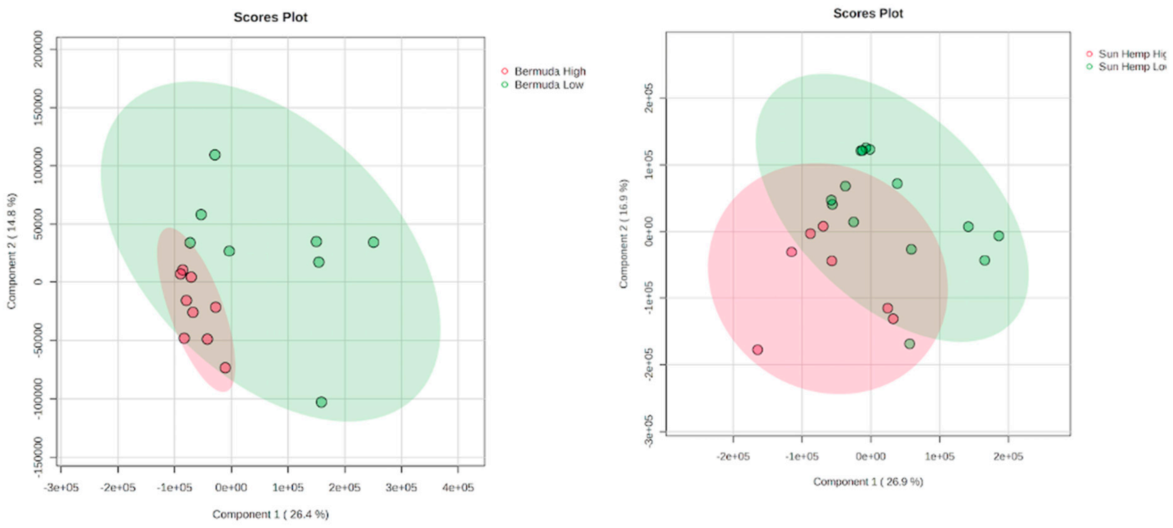

Supplement: Supplementary file 1 [file metabolites-15-00741-s001.zip › Supplementary figures for NMR and LC MS.pdf]
