# Supplementary figures and images for: Comparison of Metabolomic Signatures Between Low and Heavy Parasite Burden of Haemonchus contortus in Meat Goats Fed with Cynodon dactylon (Bermudagrass) and Crotalaria juncea L. (Sunn Hemp)
Source: Metabolites. 2025 Nov 14;15(11):741. doi: 10.3390/metabo15110741 (PMC12654777; doi:10.3390/metabo15110741)

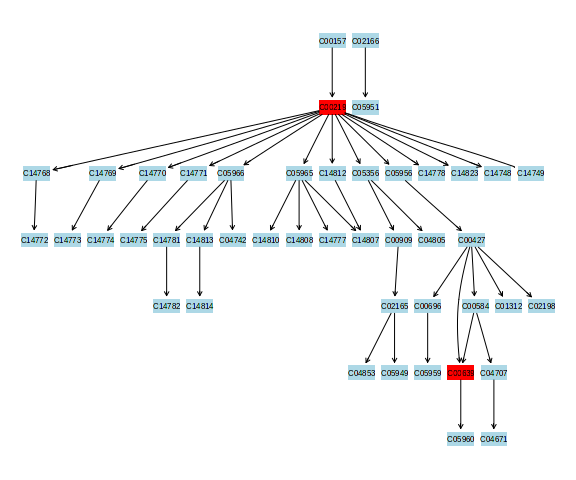

Supplement: Supplementary file 1 [file metabolites-15-00741-s001.zip › LC_MS/LC_MS/lcms bermuda pathway_new/Arachidonic acid metabolism.png]

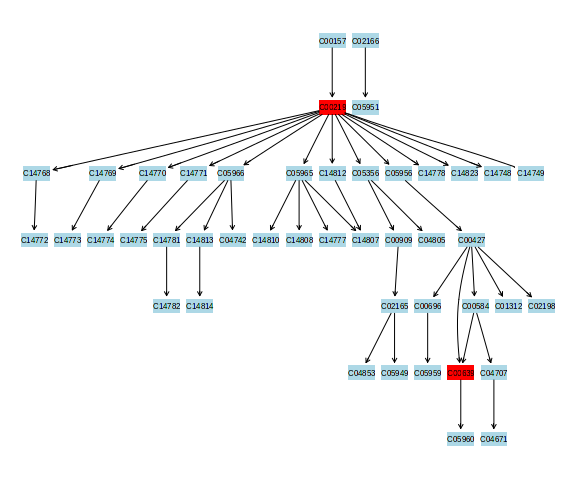

Supplement: Supplementary file 1 [file metabolites-15-00741-s001.zip › LC_MS/LC_MS/lcms bermuda pathway_new/crop1753467523752.png]

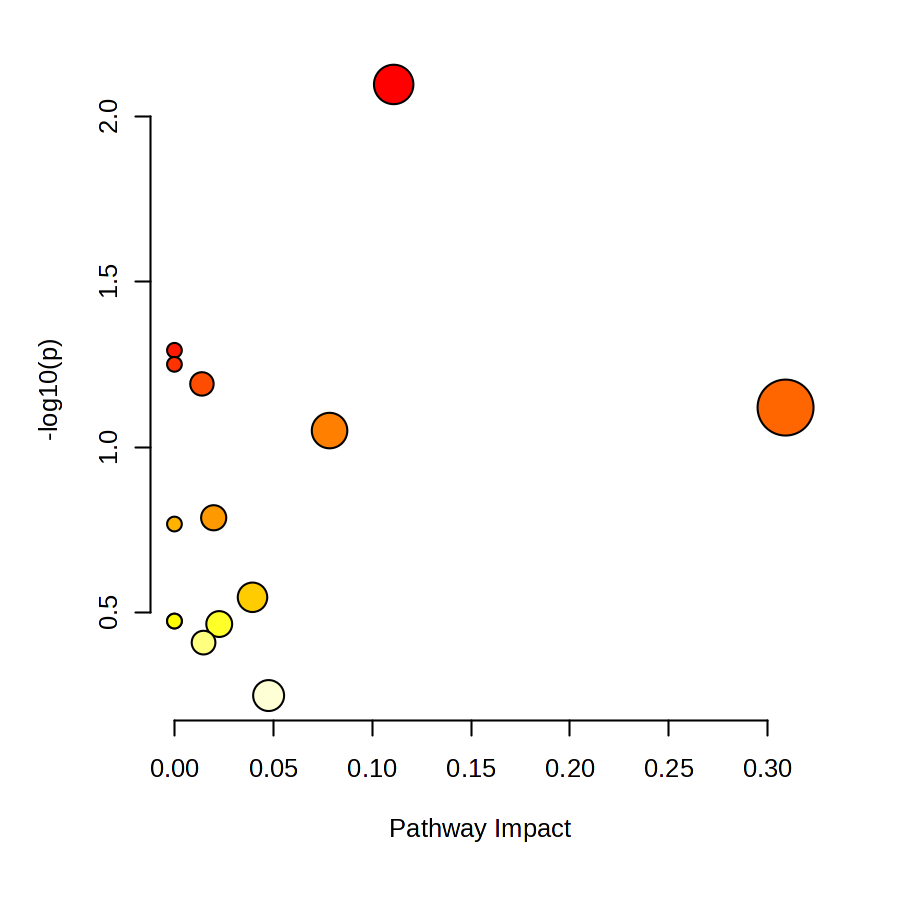

Supplement: Supplementary file 1 [file metabolites-15-00741-s001.zip › LC_MS/LC_MS/lcms bermuda pathway_new/path_view_0_dpi150.png]

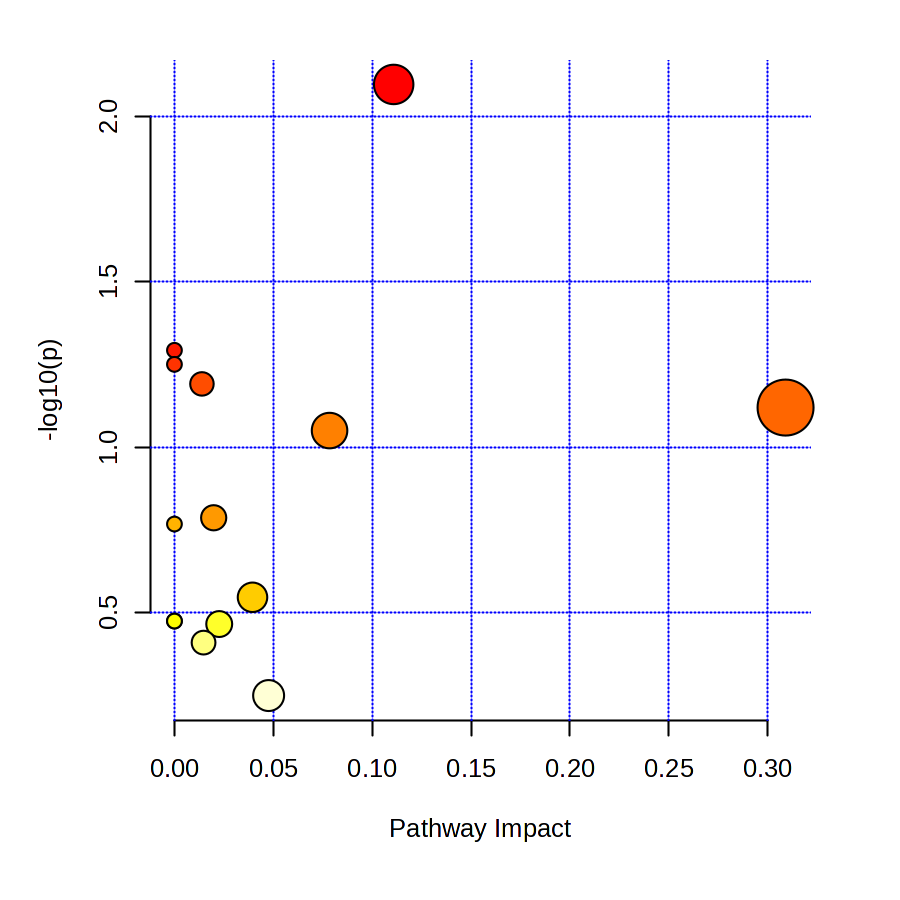

Supplement: Supplementary file 1 [file metabolites-15-00741-s001.zip › LC_MS/LC_MS/lcms bermuda pathway_new/path_view_1_dpi150.png]

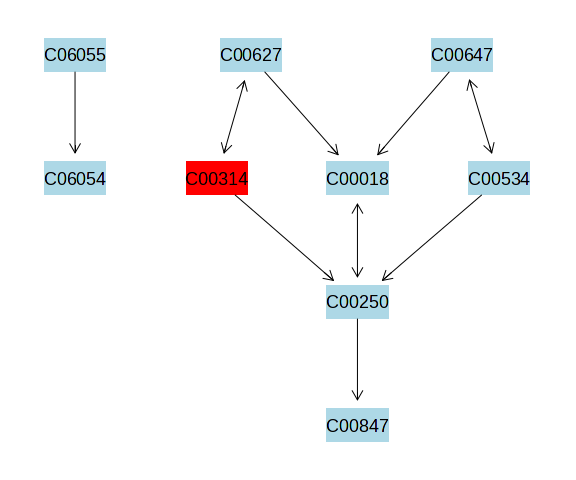

Supplement: Supplementary file 1 [file metabolites-15-00741-s001.zip › LC_MS/LC_MS/lcms bermuda pathway_new/Vitamin B6 metabolism.png]

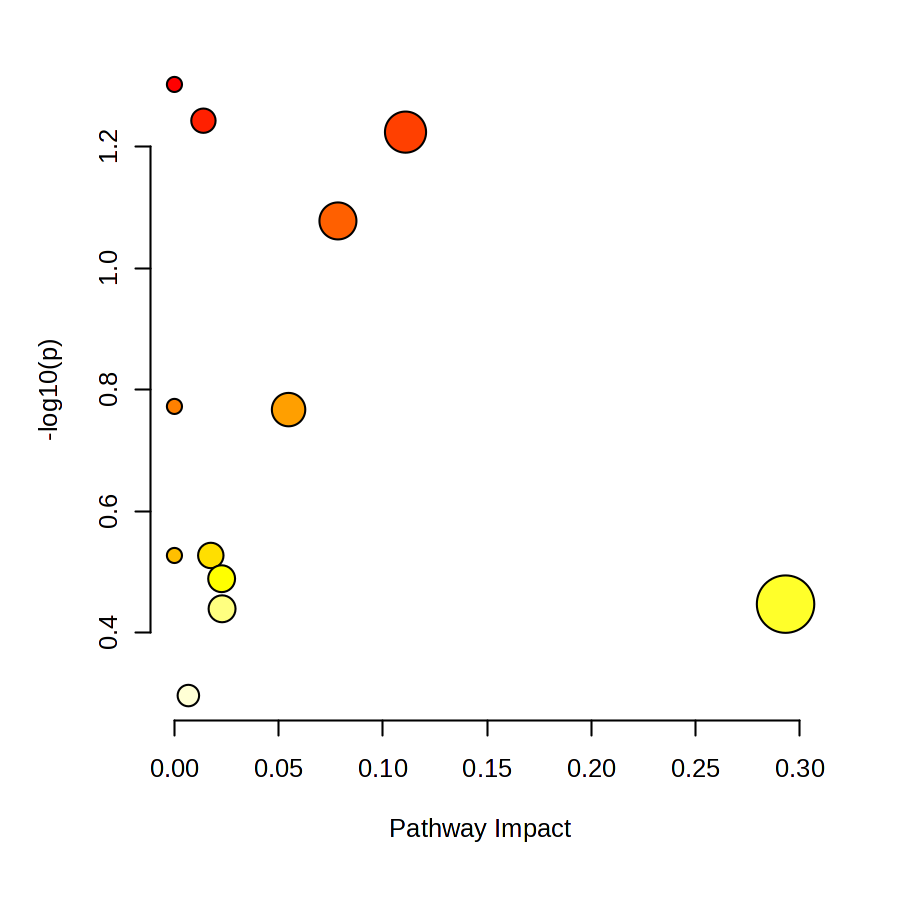

Supplement: Supplementary file 1 [file metabolites-15-00741-s001.zip › LC_MS/LC_MS/lcms sunn hemp pathway/path_view_0_dpi150.png]

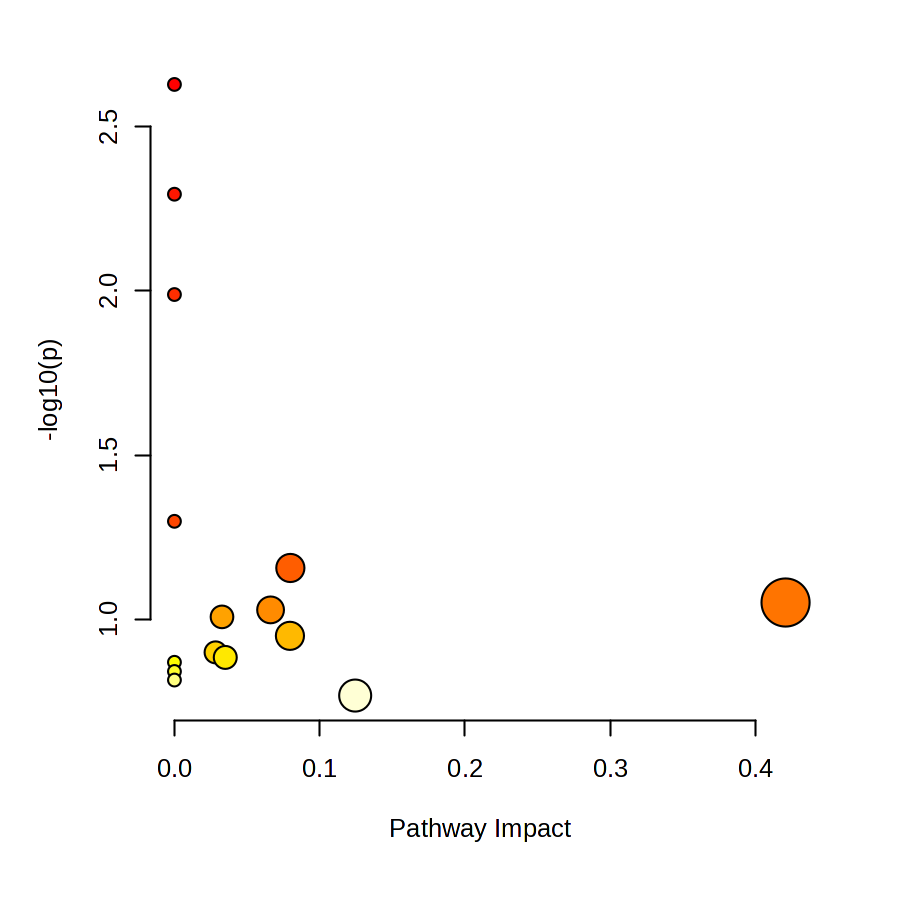

Supplement: Supplementary file 1 [file metabolites-15-00741-s001.zip › NMR/NMR/pathway result bermuda nmr/path_view_0_dpi150.png]

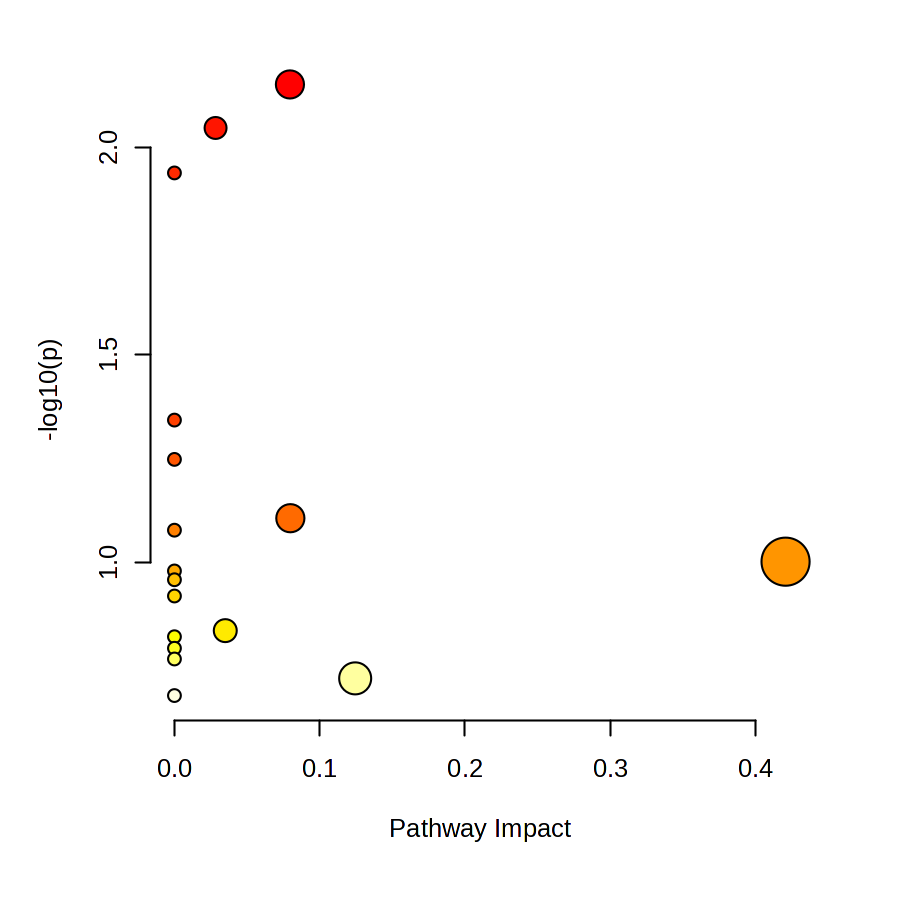

Supplement: Supplementary file 1 [file metabolites-15-00741-s001.zip › NMR/NMR/Sunn Hemp NMR pathway result/path_view_0_dpi150.png]
